# Proposing a Neurotropic Etiology for Acute Posterior Multifocal Placoid Pigment Epitheliopathy and Relentless Placoid Chorioretinitis

**DOI:** 10.3389/fopht.2021.802962

**Published:** 2022-01-10

**Authors:** Paul J. Steptoe, Ian Pearce, Nicholas A.V. Beare, Sreekanth Sreekantam, Bashar R. Mohammed, Robert J. Barry, Laura R. Steeples, Alastair K. Denniston, Philip I. Murray

**Affiliations:** ^1^ Princess Alexandra Eye Pavilion, Edinburgh, United Kingdom; ^2^ St. Paul’s Eye Unit, Liverpool University Hospitals National Health Service (NHS) Foundation Trust, Liverpool, United Kingdom; ^3^ Department of Eye and Vision Science, Institute of Life Course and Medical Sciences, University of Liverpool, Liverpool, United Kingdom; ^4^ Birmingham and Midland Eye Centre, Birmingham, United Kingdom; ^5^ Institute of Clinical Sciences, College of Medical and Dental Sciences, University of Birmingham, Birmingham, United Kingdom; ^6^ Manchester Royal Eye Hospital, Manchester University Hospitals NHS Foundation Trust, Manchester Academic Health Science Centre, Manchester, United Kingdom; ^7^ Faculty of Biology, Medicine & Health, University of Manchester, Manchester, United Kingdom; ^8^ Institute of Inflammation and Ageing, College of Medical and Dental Sciences, University of Birmingham, Birmingham, United Kingdom; ^9^ Ophthalmology Department, University Hospital Birmingham NHS Foundation Trust, Birmingham, United Kingdom; ^10^ Centre for Rare Diseases, University Hospital Birmingham NHS Foundation Trust, Birmingham, United Kingdom

**Keywords:** acute posterior multifocal placoid pigment epitheliopathy, relentless placoid chorioretinitis, von Szily model, axonal spheroids, Henle fiber layer, Neurotropic virus

## Abstract

**Purpose:**

To reassess the underlying pathophysiology of acute posterior multifocal placoid pigment epitheliopathy (APMPPE) and relentless placoid chorioretinitis (RPC) through comparison with the non-inoculated eye of the von Szily animal model of neurotropic viral retinal infection.

**Methods:**

Narrative review.

**Results:**

Literature reports of isolated neurotropic viral entities and rising serological viral titers in APMPPE after presentation support a potential direct infective etiology. In general, viral transport along axons results in mitochondrial stasis and disruption of axoplasmic flow. Clinical manifestations of axoplasmic flow disruption in APMPPE/RPC may signify the passage of virus along the neuronal pathway. From a case series of 11 patients, we demonstrate a timely, spatial, and proportional association of optic disc swelling with APMPPE lesion occurrence. Signs within the inner retina appear to precede outer retinal lesions; and acute areas of outer nuclear layer (ONL) hyperreflectivity appear to be the result of coalescence of multiple hyperreflective foci resembling axonal spheroids (which occur as a consequence of axoplasmic disruption) and follow the Henle fiber layer neurons. Underlying areas of retinal pigment epithelium (RPE) hyper-autofluorescence follow ONL hyperreflectivity and may signify localized infection. Areas of apparent choriocapillaris hypoperfusion mirror areas of RPE/Bruch’s membrane separation and appear secondary to tractional forces above. Increases in choroidal thickness with lesion occurrence and focal areas of choriocapillaris hypoperfusion are observed in both APMPPE/RPC and the von Szily model.

**Conclusions:**

The neurotrophic infection model provides significant advantages over the existing primary choriocapillaris ischemia hypothesis to account for the range of imaging signs observed in APMPPE and RPC.

## Introduction

The pathogenesis of herpes simplex virus (HSV) keratitis illustrates how the nerve supply to ocular tissues can opportunistically be utilized by neurotropic pathogens as a means of transit to target tissues from sites of latency within central nervous system (CNS) nuclei. While the pathogenesis for HSV corneal infection is well established, the potential for retinal infection *via* the ganglion cell axons is less clinically recognized yet equally demonstrated in animal models (1, 2).

**Figure 1:**
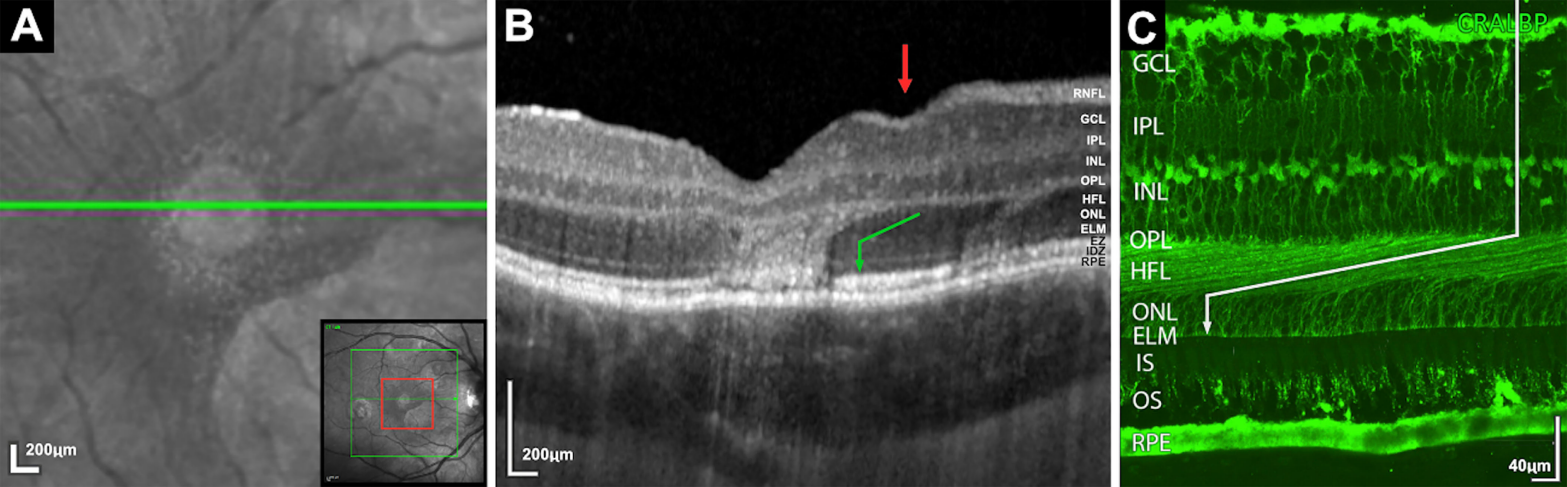
**(A)** Magnified infrared image demonstrating foveal lesion (increased infrared signal) with encircling speckled halo of increased infrared dots. Bottom right insert, red square demonstrates the area of magnification. **(B)** Spectral-domain optical coherence tomography corresponding to area **(A)** Two lesions demonstrating areas of ONL hyperreflectivity with oblique margins within the Henle Fiber layer (HFL) before orientating vertically and obscuring the external limiting membrane, ellipsoid zone and interdigitation zone. Green arrow indicates HFL orientation. Red arrow demonstrates associated depression of retinal surface vertically above the descending fibers. **(C)** Human retina immunostained with antibodies against cellular retinaldehyde-binding showing the orientation of the Muller cells and cone axons which constitute the HFL. (Image C courtesy of Cuenca et al) (3).

Observations of acute lesions in acute posterior multifocal placoid pigment epitheliopathy (APMPPE) demonstrate areas of outer nuclear layer (ONL) hyperreflectivity follow the Henle fiber layer (HFL) (**Figure 1**) (4). In addition, evidence of simultaneous viral infection (5–7), and a commonly reported viral prodrome in approximately a third of patients (8), highlight the possibility of a direct viral infective etiology in what is currently regarded as a non-infective and primary ocular posterior uveitis.

In this article, we introduce the reader to the von Szily animal model of viral neurotropic retinal infection and outline the evidence to support an infective association with APMPPE and the related entity *relentless placoid chorioretinitis* (RPC). We discuss the evidence supportive of neuronal involvement in these entities and compare clinical imaging examples with the histopathological findings of neurotropic viral retinopathies from von Szily animal models to illustrate parallels between the two.

In 1924, von Szily demonstrated that following the inoculation of herpes simplex virus (HSV) into the ciliary body dialysis cleft of one eye in rabbits, a delayed retinitis in the contralateral, non-inoculated eye occurs (9). This phenomenon has been reproduced with other neurotropic viruses (2, 10) and replicated in mouse models with contralateral retinopathies produced following anterior chamber (11, 12) and vitreous chamber inoculation (13). While APMPPE is commonly a bilateral condition, bilateral neurotropic retinopathies were produced following intracerebral viral inoculation (14) or in immunosuppressed (15–17) or high viral dose inoculation of the von Szily model (11, 16, 18, 19). Following propagation along the parasympathetic fibers of the oculomotor nerve which supply the iris and ciliary body of the inoculated eye, viral dissemination in the CNS is limited primarily to nuclei of the visual system (20). Viral transmission from the CNS to the retina of the contralateral eye occurs *via* retrograde axonal transport through the optic nerve along the endocrine-optic pathway between the retina and the suprachiasmatic nucleus of the hypothalamus (20).

Whilst the von Szily animal model often results in a severe, full-thickness retinal necrosis to varying extents in the non-inoculated eye (depending on a combination of host immunity vs viral virulence), it is the early morphological changes in the non-inoculated eye, before this endpoint which offer the basis for our comparison, providing an endogenous viral entry pathogenesis, free from injection artifact. For clarity, in referring to the von Szily model from this point onwards, it is the observations from the non-inoculated eye we are referring to.

## Potential for Infective Etiology

Since its original description (21), multiple infective etiologies with known neurotropism have been associated with APMPPE including adenovirus (5, 6), measles (22), mumps (23), dengue virus (24), lyme disease (25, 26), and tuberculosis (21, 27). While viral neurotropism is widely known, the ability of bacteria such as *Mycobacterium tuberculosis* to also possess neurotropic capability is a relatively new discovery (28). The association of adenovirus Type 5 with APMPPE is particularly supportive due to several factors. Azar et al. reported a serological rise in adenovirus type 5 antibody titer and isolated adenovirus type 5 from two sources (throat swab and tonsil biopsy) during concurrent APMPPE (5), while Thomson and Roxburgh isolated Adenovirus Type 5 from a conjunctival swab following the development of a follicular conjunctivitis post-prednisolone treatment for APMPPE (6). The potential association of adenovirus is also supported by reports of preceding upper respiratory tract infections (8) and a higher incidence of HLA-B7 in APMPPE patients (relative risk 3.38) (29). HLA-B7 is known to confer an additional risk to adenovirus secondary to its increased affinity to the adenoviral 19K protein in comparison to other alleles (30). The adenoviral 19K protein retains HLA class I molecules in the endoplasmic reticulum, thus preventing the presentation of viral antigenic peptides at the cell surface and consequently cytotoxic T cell recognition of adenovirus-infected cells is averted (30). The reported case of 3 family members with the HLA-B7 and HLA-DR2, two of which developed optic neuritis, and one APMPPE within 4 months is also suggestive of an infective etiology (31). CNS manifestations associated with APMPPE (8) and RPC (32) and pleocytosis occurrence (8, 32) (most commonly associated with CNS viral infection) (33) also suggest a potential CNS origin for APMPPE/RPC in keeping with the von Szily model.

The occurrence of APMPPE post influenza vaccination has also been reported (34, 35). While the specific vaccine type (egg-based, cell-based or recombinant) is not specified in these case reports, many strains of the influenza virus possess neurotropic capabilities (36, 37). Influenza virus has also been associated with cases of acute macula neuroretinopathy which shares a number of imaging characteristics with APMPPE (38). In addition, the potential for recombinant vaccines to exhibit an unanticipated neurotropism and have the capability to cause direct ocular infection has been demonstrated in the Ebola vaccine (rVSVΔG-ZEBOV-GP) (39).

## Evidence for Neuronal Involvement

### Optic Nerve Involvement

**Figure 2:**
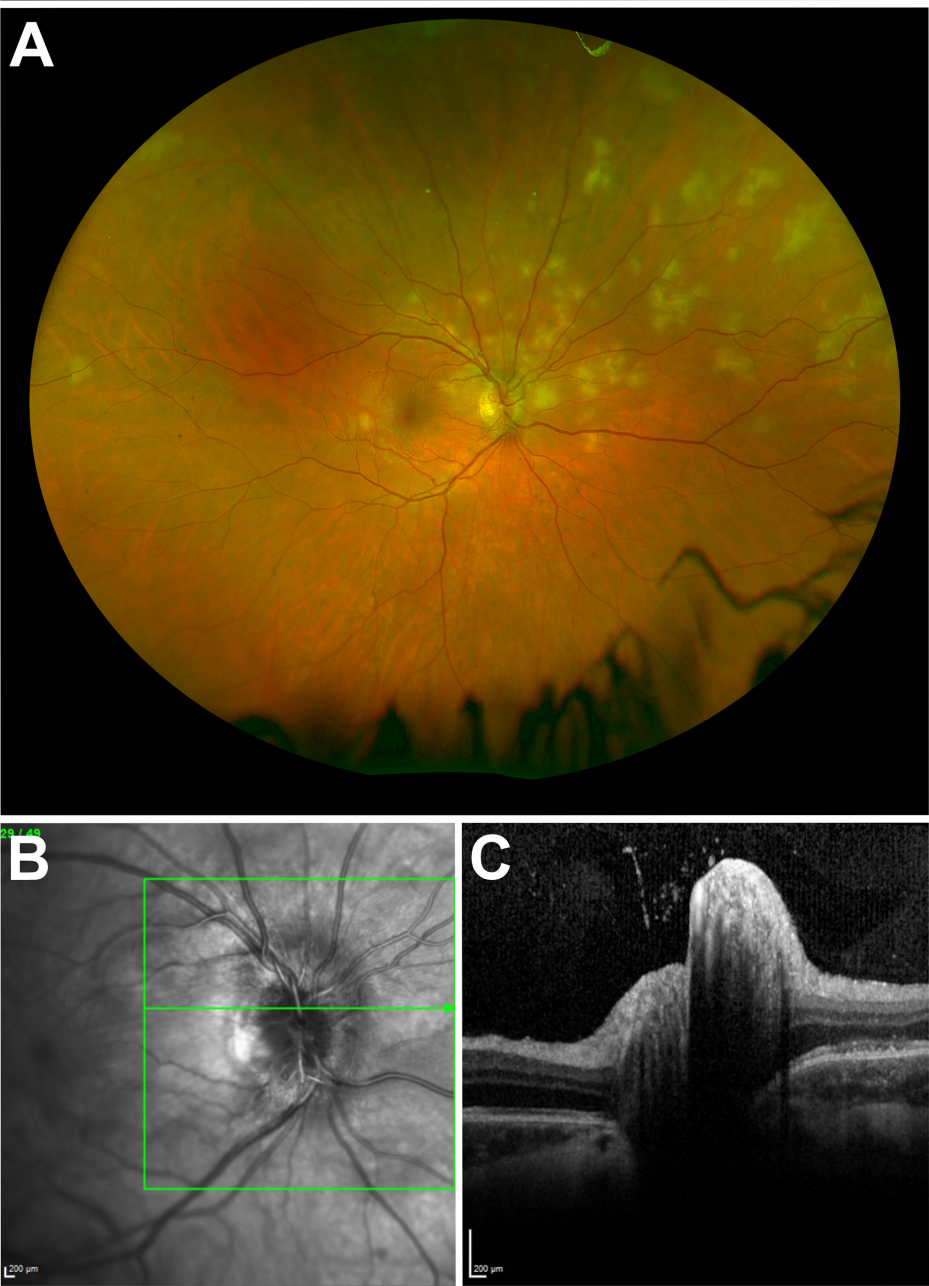
**(A)** Ultra-widefield fundus imaging demonstrating lesions predominantly clustered in the superior nasal retinal quadrant. **(B)** Infrared image of the right eye optic disc demonstrating corresponding sectoral disc edema. **(C)** Corresponding right eye optic disc spectral-domain optical coherence tomography appearance.

The observation of optic disc edema (and by definition, a disruption of axoplasmic flow) with cases of APMPPE/RPC has been frequently reported (8, 40) yet received little consideration. In the following examples, we demonstrate how this is both timely, spatially, and proportionally associated. Increases in optic disc edema (and secondary increases in retinal vessel diameter and tortuosity) can be observed to occur concurrently with the presence of new retinal lesions (**Supplementary Video 1**) and coincide with episodes of lesion recurrence after periods of quiescence (**Supplementary Video 2**). A reversible relationship between lesions and disc edema is also evident as corresponding reductions in retinal vessel tortuosity and optic disc edema are observed following the resolution of acute lesions (**Supplementary Video 3**). Spatially, where lesions appear predominately limited to a single retinal sector, a corresponding sectoral optic disc edema can be observed (**Figure 2**) (40). Finally, the extent of disc edema appears proportional to the number of lesions in bilateral cases (**Supplementary Figure 1**).

In the von Szily model, virions initially enter the eye *via* axonal transport along the ganglion cell axons (20). The pathophysiology of viral axonal transport provides a potential explanation for optic disc edema associated with lesion occurrence. Since no viral genome encodes molecular motors, viruses must encode adaptor or modifying gene products to repurpose neuronal components to enable their axonal transport (41). One such means is the ‘hijacking’ of the mitochondria motility protein, kinesin-1 (42). While enabling the propagation of virions along the neuronal microtubule, it does so at the expense of mitochondrial motility (42), leading to stasis and accounting for the disruption of axoplasmic flow with simultaneous lesion occurrence (**Supplementary Video 4**) **(** 43).

### Henle Fiber Layer Involvement

**Figure 3:**
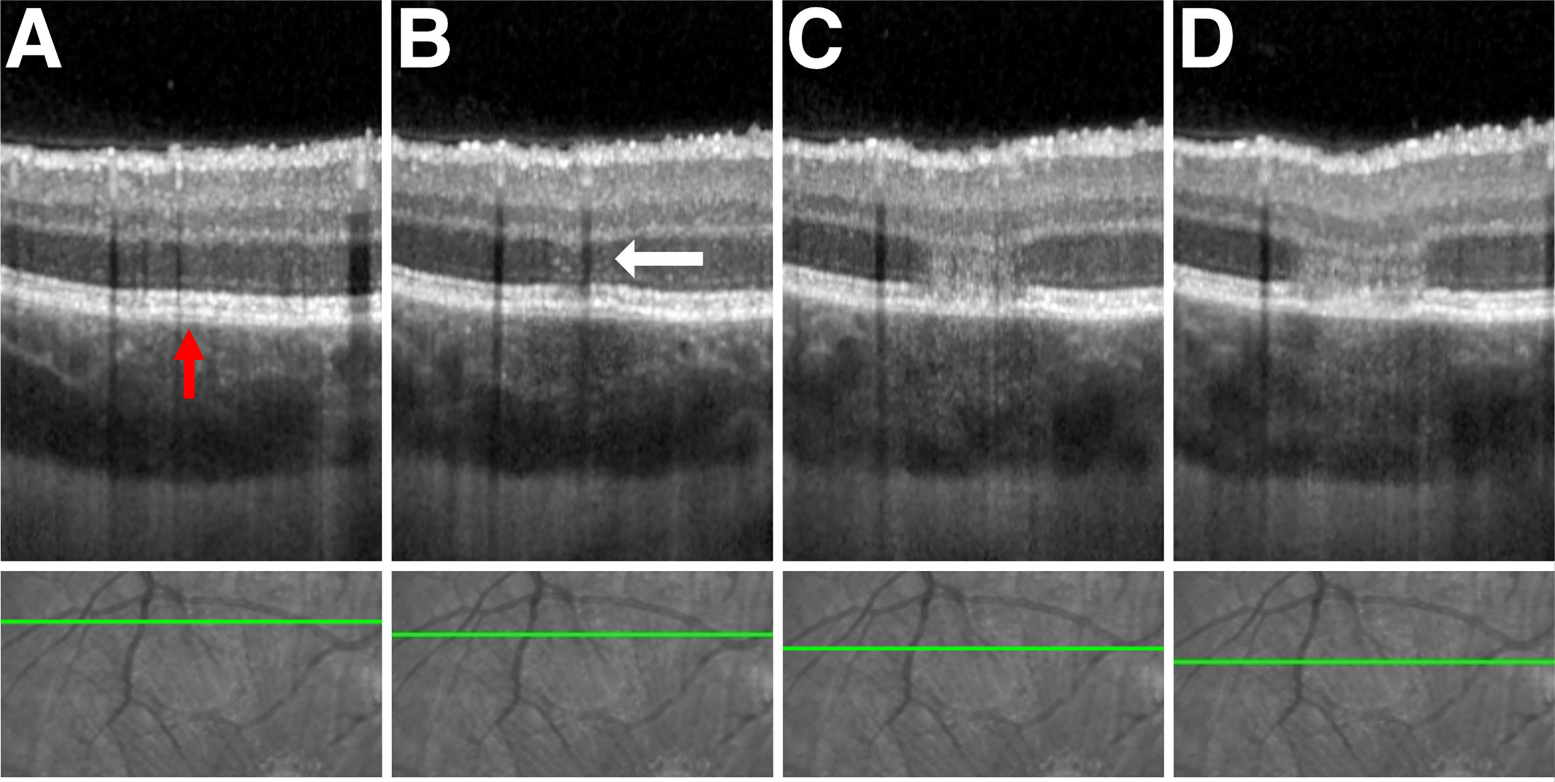
Advancing phases of APMPPE lesion from lesion margin to lesion center. Upper row – spectral-domain optical coherence tomography (SD-OCT). Lower row - corresponding infrared fundus image. The green line denotes the scan section through the lesion with progressive slices towards the center left to right. **(A)** Superior peripheral margin of the lesion with a subtle elevation of retinal pigment epithelium (RPE) from Bruch’s membrane (BM) visible (red arrow). **(B)** Lesion peripheral edge. Multiple hyperreflective foci (white arrow) seen within the outer nuclear layer (ONL). Ellipsoid zone (EZ) and RPE hyperreflective bands are still visible. **(C)** Confluent hyperreflectivity of the ONL, loss of the EZ and RPE band hyperreflectivity, and marginal RPE/BM separation. **(D)** Broadening column of ONL hyperreflectivity with subluxation of all overlying retinal layers.

**Figure 4:**
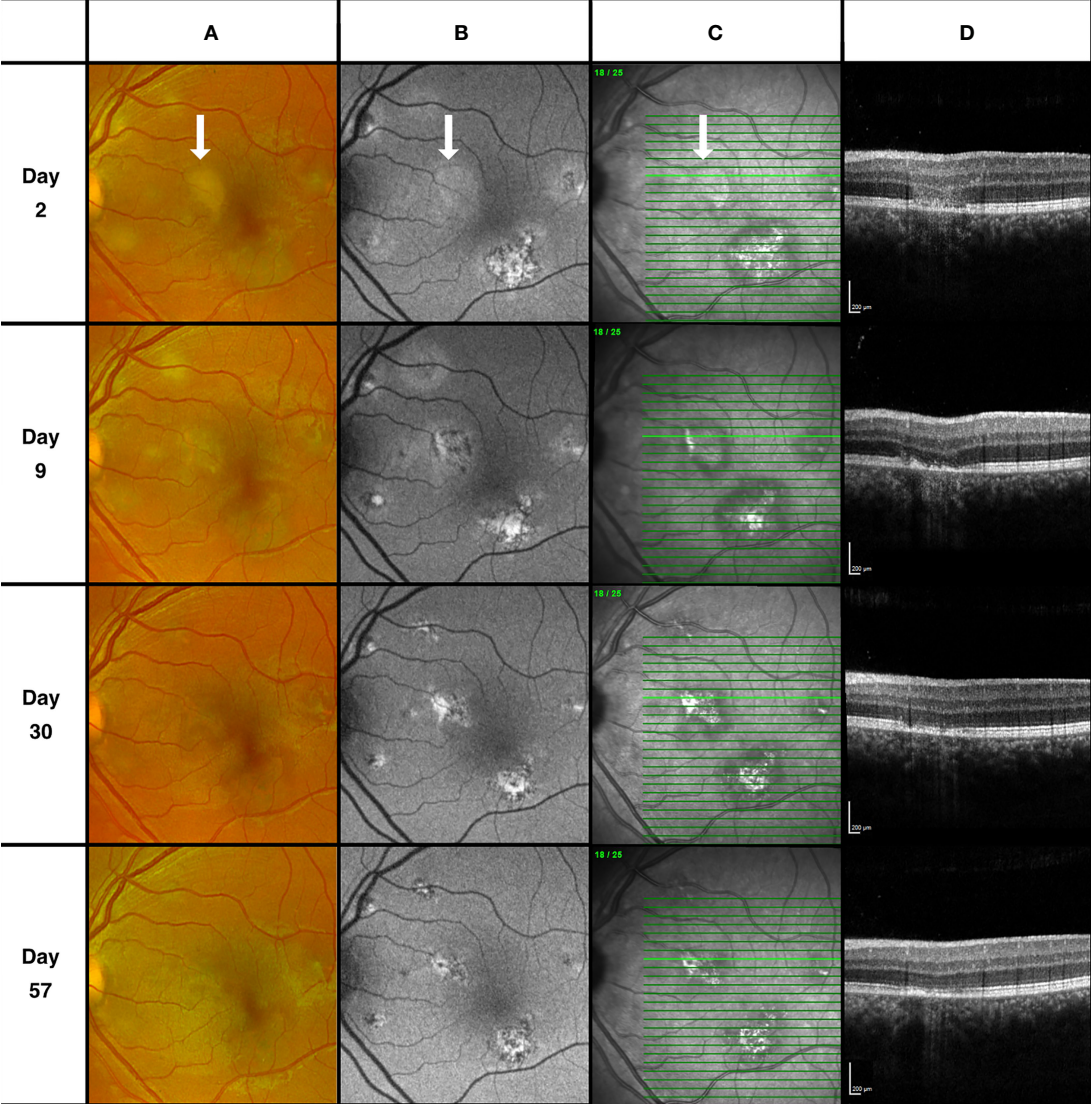
Descending retinal structural loss. Column **(A)** Fundus scanning laser ophthalmoscopy. Column **(B)** Fundus autofluorescence (FAF) imaging. Column **(C)** Fundus infrared (IR) imaging. Column **(D)** Spectral-domain optical coherence tomography (SD-OCT). Day 2, Retinal pigment epithelium (RPE) band on SD-OCT appears intact with overlying outer nuclear layer (ONL) hyperreflectivity. Corresponding lesion area on FAF demonstrates faint hyper autofluorescence only. RPE disturbance on SD-OCT and corresponding changes visible on FAF apparent at day 9 while the area of ONL hyperreflectivity has resolved. Appearance at day 30 and 57 demonstrate gradual restoration of the outer retinal structures.

Optical coherence tomography (OCT) observations of the ONL at the time of acute lesions provide further evidence of neuronal involvement in APMPPE/RPC. Outside of the foveal region, areas of ONL hyperreflectivity appear vertically orientated (**Figure 3**). However, within the foveal region, areas of ONL hyperreflectivity mirror the angulations of the HFL when the orientation of the OCT scan is in parallel with the circumferentially orientated HFL as seen in **Figure 1**. As the HFL is constituted of unmyelinated photoreceptor axons, this suggests areas of ONL hyperreflectivity represent a change along these nerve axons. Given that, expanding APMPPE lesions do so from the center outwards, fine OCT slices through a lesion from the margin towards the center provide a timeline of lesional evolution with the earliest changes at the margin and oldest towards the center (**Figure 3**). This evidence combined with evidence obtained from consecutive frequent OCT imaging suggest that areas of confluent ONL hyperreflectivity are preceded by, and therefore constituted of multiple, individual speckled hyperreflective dots (**Figure 3**). Since the ONL hyperreflective dots are occurring along the path of the HFL (photoreceptor axons and Muller cells), these may represent the formation of axonal spheroids (accumulations of multivesicular bodies and disorganized cytoskeletal elements) which occur in an asynchronous formation as a consequence of a disruption of axoplasmic flow along an axon (44). We previously discussed the potential mechanism by which viral nerve infection can lead to a disruption of axoplasmic flow and therefore the formation of axonal spheroids may signify the passage of virus to the photoreceptors and RPE. The hyper-autofluorescence signal observed from the RPE may therefore be a secondary response to RPE infection (**Figure 4**) The fact that the ONL reflectivity resolves back to its usual hyporeflective appearance following the acute lesion phase mirrors the behavior of axonal spheroids which can resolve once axoplasmic flow is restored (45).

## Lesion Evolution and the von Szily Model

**Figure 5:**
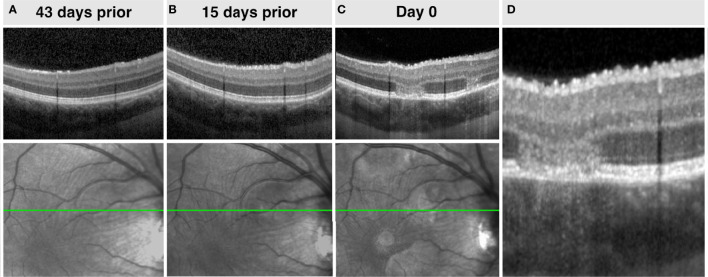
Top row **(A-C)**. Right eye spectral-domain optical coherence tomography (SD-OCT) demonstrating increasing retinal surface undulations and hyperreflective foci and stable choroidal appearance before the occurrence of APMPPE lesions. Bottom row **(A-C)**. Corresponding sequential infrared fundus imaging preceding the occurrence of lesions demonstrating retinal undulations. **(D).** Magnified SD-OCT of image C.

Areas of choriocapillaris ischemia are regarded by many to be the primary disorder in APMPPE/RPC (46). However, in the von Szily model, viral propagation enters the eye *via* the ganglion cell axons before descending vertically either partially or completely to the photoreceptor layer (20). Therefore, changes within the retinal nerve fiber layer (RNFL) before the occurrence of outer retinal lesions, would provide additional supporting evidence that APMPPE/RPC may share a neurotropic pathogenesis. **Figure 5** provides an example of such an occurrence in a case of RPC with a 43-day interval before the second eye involvement. Although the appearance of retinal folds was observed in the non-involved eye at the time of presentation, multiple hyperreflective retinal surface foci and saw-tooth like distortions of the RNFL with or without hyperreflective apices were observed on OCT at 15 days before the more typical outer retinal lesions appeared and symptoms occurred. These signs were transient and resolved following the early presentation.

**Figure 6:**
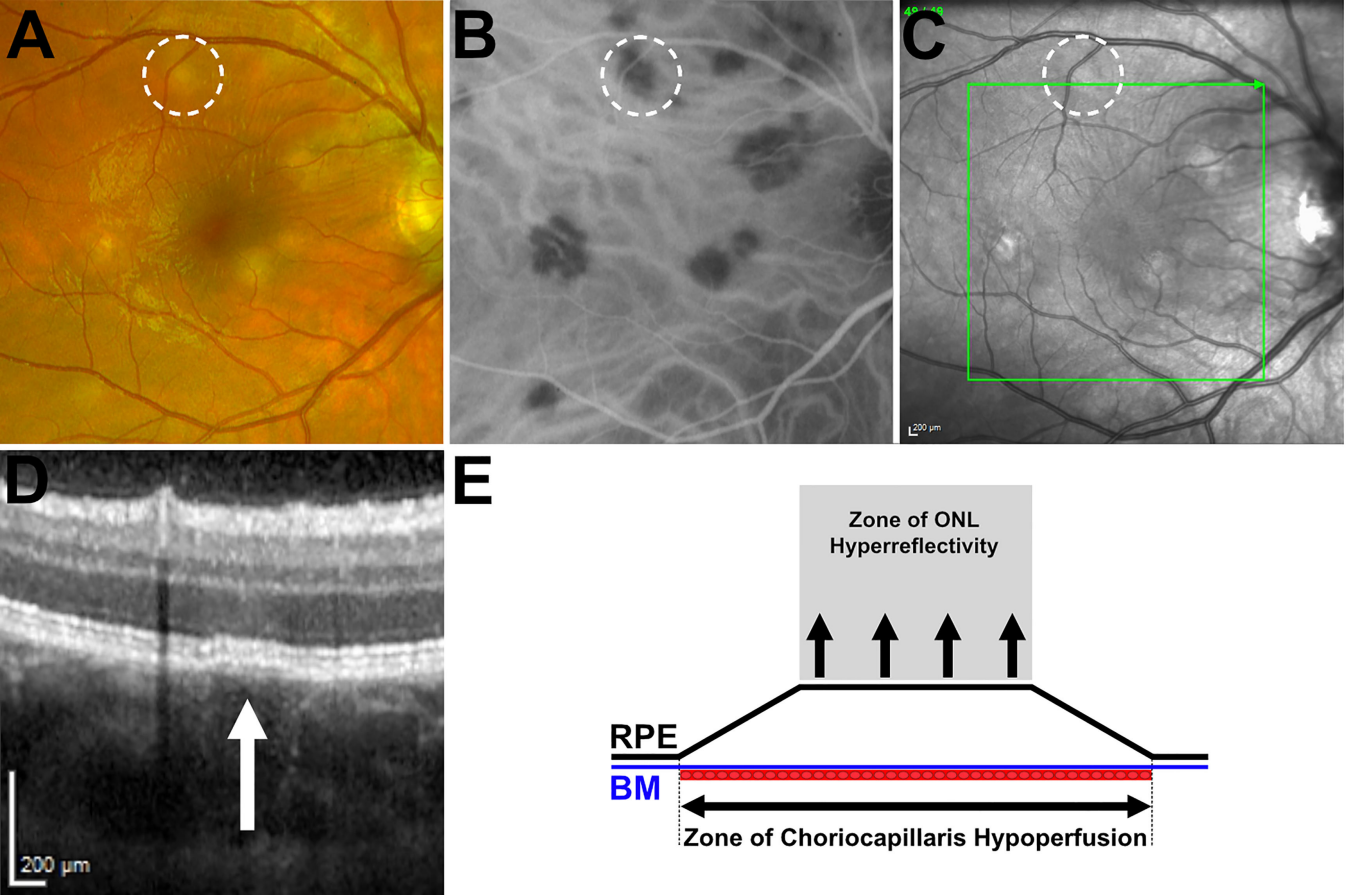
Right eye, multimodal imaging comparison of an early lesion. **(A)** Fundus scanning laser ophthalmoscopy demonstrating characteristic creamy yellow lesion appearance. **(B)** Indocyanine green angiography (ICGA) demonstrating area of hypofluorescence associated with the lesion. **(C)** Infrared (IR) fundus image. Green arrow denotes orientation of spectral-domain optical coherence tomography (SD-OCT) scan reference line; **(D)** SD-OCT of early lesion appearance. White arrow indicates area of retinal pigment epithelium (RPE) and Bruch’s membrane separation with a plateau top configuration. **(E)** Diagrammatic representation of the outer retina highlighting the area of choriocapillaris hypoperfusion in relation to the area of retinal pigment epithelium/Bruch’s membrane separation and the corresponding zone of outer nuclear layer hyperreflectivity.

The asynchronous timing of lesions and short intervals between repeat examinations provides evidence to suggest that areas of choriocapillaris hypoperfusion appear in conjunction with the presence of focal, subtle elevations of the retinal pigment epithelium (RPE) with an apparent separation from Bruch’s membrane (BM). This enables the discrimination of both, ordinarily indistinguishable hyperreflective structures on OCT (**Figure 6** **;** **Supplementary Figure 2**). The classical, creamy colored APMPPE lesions with indistinct margins on color imaging also appear to correlate to the area of RPE/BM separation and may be secondary to the contents within a cavity formed between the two structures. A corresponding upward deviation of the overlying interdigitation zone (IDZ), ellipsoid zone (EZ), and external limiting membrane (ELM) may also be seen. The appearance of areas of RPE/BM separation is typically transient and often becomes indiscernible with flattening and development of areas of ONL hyperreflectivity and progressive structural loss. In contrast, the duration of areas of choriocapillaris hypoperfusion and typically observed recovery usually occur over a longer time frame, in the order of weeks to months, depending on lesion size (**Supplementary Figure 3**).

The appearance of areas of RPE/BM separation (with a seemingly nondisplaced BM, yet elevated, plateau topped RPE) with no apparent tissue loss and no intense hyper-autofluorescence (at this stage of lesion evolution) provides an insight into the forces leading to its morphological appearance. Any force from beneath the BM would result in a secondary upward deviation of both BM and the RPE, while if the appearance were *solely* secondary to a fluid accumulation between the two structures, a resultant dome-shaped elevation of the RPM may be expected. Instead, the plateau topped, elevated RPE appearance seen in **Figure 6** with clear marginal angulations tapering down to re-join the BM, appears more consistent with a tethering effect from a secondary tractional force in the overlying retina. In parallel with the von Szily model, Holland et al. also noted early disruption of the outer retinal structures (in the absence of viral particles) (11). They hypothesized that following release from the ganglion cells, viral infection of the adjacent Muller cells occurs (the supporting retinal neuroglia). Unlike neurons which are only susceptible at their synaptic endings, the entire surface of neuroglia cells such as the Muller cell is susceptible to viral penetration (11). Destruction of the Muller cells which provide the architectural support structure to the retina, extending from the internal limiting membrane to the external limiting membrane may potentially account for the upward traction on the RPE-transmitted either *via* the interdigitations between the photoreceptors and RPE villi or to the ELM itself.

The mechanism of how changes within the choriocapillaris result as a consequence of a separation between the RPE and BM is unclear but focal loss of the choriocapillaris associated with lesions has also been observed in the von Szily model (10). Given the choriocapillaris is responsible for vascular perfusion of the RPE, it may occur as a direct consequence of anatomical trauma caused by the mechanical separation of the two structures or as a secondary vasoconstrictive response.

Observations of increases in choroidal thickness with lesion occurrence are also paralleled in both entities (4, 10, 11) (**Supplementary Video 5**). In a rabbit model utilising a fluorescein-labelled antibody technique, Petite et al. reported “at no time were viral antigens identified in the choroid of these eyes although it was always greatly thickened and heavily infiltrated with inflammatory cells when uveitis was present” (13). Similarly, histological evidence of an increase in choroidal thickness with an increase in polymorphonuclear leukocytes in response to the outer retinal infection was noted by Holland et al (11).

While our observations have focused primarily on APMPPE and RPC, given the parallels in many of the signs and symptoms associated with related conditions our hypothesis may have wider implications for other entities. Like APMPPE, a viral prodrome is frequently reported in both multiple evanescent white dot syndrome (MEWDS) (47), Vogt-Koyanagi-Harada syndrome (VKH) (48) and acute macular neuroretinopathy (AMN) (49). While both AMN and MEWDS have been associated with acute viral infections specifically (Influenza virus (38) and Epstein-Barr virus (EBV) (50) respectively), EBV has been isolated by polymerase chain reaction (PCR) from the vitreous (51) and cerebrospinal fluid (52) in patients with VKH.

As we have demonstrated increases in optic disc edema and choroidal thickness which acute activity in APMPPE/RPC, similar findings have been reported in MEWDS (53, 54) VKH (55, 56), AMN (57, 58) and punctate inner choroidopathy (PIC) (59, 60).

Areas of ONL hyperreflectivity following the HFL, identical to those demonstrated in APMPPE (**Figure 1**) are also seen in acute macula neuroretinopathy (49) and hyperreflective dots following the HFL have been demonstrated in MEWDS (61). While the appearance of bacillary layer detachments is most commonly associated with cases of VKH, the appearance has also been reported in cases of APMPPE (4, 62–64) and observed in our series (**Supplementary Figure 4**). As we have hypothesised that areas of hypofluorescence on indocyanine green angiography occur as a consequence of RPE-BM separation (**Figure 6**), areas of RPE-BM separation can also be observed in early bacillary layer detachments (**Supplementary Figure 4**). Our observations of inner retinal changes preceding outer retinal signs in APMPPE (**Figure 3**) have also been mirrored in VKH, with internal limiting membrane folds being reported to precede bacillary layer detachments (65).

From our cases series, we have observed how an initial presentation classically resembling APMPPE, can progress to resemble a phenotype in keeping with Birdshot chorioretinopathy (**Supplementary Figure 5**). Although rare, cases of depigmented chorioretinal lesions, resembling Birdshot chorioretinopathy have been shown to occur following Varicella-Zoster Virus infection (66, 67).

Beyond the spectrum of the white dot syndromes, a neurotropic means of viral transmission to the retina has been hypothesised in both Ebola Virus Disease retinopathy (68, 69) and West Nile Virus associated retinopathy (70, 71) based on the distribution of peripapillary lesions following the ganglion cell axon anatomical pathway. While Dengue virus, although not known for its neurotropism, has also been attributed to several cases of APMPPE (24).

## Limitations

Our study is limited by its small sample and retrospective design. Our evidence of RNFL disturbances preceding lesion occurrence is limited to two cases due to the chance scenarios of sequential eye involvement permitting contralateral, pre-symptomatic macula imaging. Secondly, our conclusions are the summation of imaging collated from clinical reviews at differing intervals, varying imaging protocols, and devices due to a combination of availability and clinician preferences at the study sites.

## Directions of Future Studies

The utilisation of matched, high resolution enhanced depth imaging of the macula combined with OCT-angiography and OCT of the optic disc repeated at frequent interval during the rapidly evolving acute phase of lesion evolution would enable our hypothesis of retinal sequelae to be tested.

Given that more than half of patients with APMPPE will exhibit cells in the vitreous or aqueous (8) the potential for viral detection from ocular fluids utilising PCR or metagenomic deep sequencing (72) would provide definitive evidence for a direct infective role.

## Conclusions

The pathogenesis of the non-inoculated eye in the von Szily or neurotrophic infection model provides a conceptual framework in which to interpret the clinical signs observed in cases of APMPPE/RPC. Given the significant parallels with associated viral entities, neuronal involvement, destructive descending sequelae and shared choroidal responses, the neurotrophic infection model has significant advantages over the existing primary choriocapillaris ischemia hypothesis to account for the range of signs observed in APMPPE and RPC.

## Data Availability Statement

The raw data supporting the conclusions of this article will be made available by the authors, without undue reservation.

## Ethics Statement

Ethical review and approval was not required for the study on human participants in accordance with the local legislation and institutional requirements. The patients/participants provided their written informed consent to participate in this study. Written informed consent was obtained from the individual(s) for the publication of any potentially identifiable images or data included in this article.

## Author Contributions

All authors listed have made a substantial, direct, and intellectual contribution to the work and approved it for publication.

## Conflict of Interest

The authors declare that the research was conducted in the absence of any commercial or financial relationships that could be construed as a potential conflict of interest.

## Publisher’s Note

All claims expressed in this article are solely those of the authors and do not necessarily represent those of their affiliated organizations, or those of the publisher, the editors and the reviewers. Any product that may be evaluated in this article, or claim that may be made by its manufacturer, is not guaranteed or endorsed by the publisher.

## Supplementary Material

Supplementary Figure 1 Disc edema proportional to lesion quantity. **(A).** Right eye ultra-widefield fundus imaging, 74 days post symptom onset. Comparatively fewer lesions than the left eye seen in ultra-wide field fundus image D. **(B).** Right eye optic disc spectral-domain optical coherence tomography (SD-OCT) demonstrating optic disc edema but significantly less edema in comparison to the left eye **(E, F)**. **(C).** Right eye optic disc infrared (IR) image. **(D).** Left eye ultra-widefield fundus image. **(E).** Left eye optic disc IR image. **(F).** Left eye optic disc SD-OCT.

Supplementary Figure 2 Multimodal imaging comparison of early lesions. **Column A,** Fundus scanning laser ophthalmoscopy appearance. **Column B**, Corresponding hypofluorescence seen on indocyanine green angiography. **Column C,** Infrared fundus imaging. **Column D,** Spectral-domain optical coherence tomography (SD-OCT) demonstrating acute lesions with retinal pigment epithelium and Bruch’s membrane separation. White circles indicate the lesion location on comparative modalities. White arrows indicate location of lesions on SD-OCT.

Supplementary Figure 3 APMPPE lesion recovery. **(A).** Swept-source optical coherence tomography (SS-OCT) of acute lesion with outer nuclear layer (ONL) hyperreflectivity. **(B).** Corresponding en face OCT angiography (OCTA) demonstrating associated choriocapillaris hypoperfusion. **(C).** Appearance at day 13 demonstrating resolution of the ONL hyperreflectivity with loss of the ellipsoid zone (EZ) and interdigitation zone (IDZ) with a dipping of overlying structures. **(D).** Partial resolution of choriocapillaris perfusion on OCTA. **(E).** Appearance at day 203, demonstrating outer retinal structure restoration. **(F).** Corresponding improvement of choriocapillaris perfusion on OCTA. Purple line denotes lesion location on SS-OCT. Purple and turquoise intersection denote corresponding APMPPE lesion location on en face OCTA imaging.

Supplementary Figure 4 Case 4, Left eye. **(A),** Days since presentation. **(B),** Scanning laser ophthalmoscopy **(C),** Infrared fundus image. Bold green line denotes spectral-domain optical coherence tomography (SD-OCT) scan position. **(D)**, SD-OCT. Creamy colored acute lesions are seen on color fundus imaging at day 3 and fade by day 24. SD-OCT demonstrates a bacillary layer detachment of two adjacent lesions which coalesce by day 3 with underlying separation of the retinal pigment epithelium (RPE) from Bruch’s membrane (BM) observed at presentation (Red arrows). Bacillary layer detachment worsens acutely following presentation before resolving without treatment by day 24. Presenting Snellen visual acuity was 6/18 which decreased to 6/24 by day 3 but improved to 6/9 by day 24 and 6/6 by the following review at day 85.

Supplementary Figure 5 Acute posterior multifocal placoid pigment epitheliopathy (APMPPE) resembling Birdshot Chorioretinopathy, Right eye. Initial presentation demonstrated multifocal APMPPE lesions within the macula. By days 15 and 25, central lesions appear to fade as new lesions radiate towards the peripheral retina. The patient was HLA A29 negative.

Supplementary Video 1 Increases in optic disc edema and retinal tortuosity with lesion occurrence.

Supplementary Video 2 Increases in optic disc edema and retinal tortuosity with lesion recurrence after quiescence.

Supplementary Video 3 Reductions in optic disc edema and vessel tortuosity following lesion resolution.

Supplementary Video 4 Mitochondrial motion during alpha-herpes virus infection in neurons. (Video courtesy of Tal Kramer).

Supplementary Video 5 Increases in choroidal thickness with lesion occurrence.

## References

[1] Atherton SS. Acute Retinal Necrosis: Insights Into Pathogenesis From the Mouse Model. Herpes J IHMF (2001) 8:69–73. 11867023

[2] Krey H Ludwig H Rott R. Spread of Infectious Virus Along the Optic Nerve Into the Retina in Borna Disease Virus-Infected Rabbits. Arch Virol (1979) 61:283–8. doi: 10.1007/BF01315014 518303

[3] Cuenca N Ortuño-Lizarán I Pinilla I. Cellular Characterization of OCT and Outer Retinal Bands Using Specific Immunohistochemistry Markers and Clinical Implications. Ophthalmology (2018) 125:407–22. doi: 10.1016/j.ophtha.2017.09.016 29037595

[4] Mrejen S Sarraf D Chexal S Wald K Freund KB. Choroidal Involvement in Acute Posterior Multifocal Placoid Pigment Epitheliopathy. Ophthalmic Surg Lasers Imaging Retina (2016) 47:20–6. doi: 10.3928/23258160-20151214-03 26731205

[5] Azar PJ Gohd RS Waltman D Gitter KA. Acute Posterior Multifocal Placoid Pigment Epitheliopathy Associated With an Adenovirus Type 5 Infection. Am J Ophthalmol (1975) 80:1003–5. doi: 10.1016/0002-9394(75)90328-1 173190

[6] Thomson SPS Roxburgh STD. Acute Posterior Multifocal Placoid Pigment Epitheliopathy Associated With Adenovirus Infection. Eye (2003) 17:542–4. doi: 10.1038/sj.eye.6700389 12802366

[7] Li AL Palejwala NV Shantha JG O’Keefe G Lee CS Albini T. Long-Term Multimodal Imaging in Acute Posterior Multifocal Placoid Pigment Epitheliopathy and Association With Coxsackievirus Exposure. PloS One (2020) 15:e0238080. doi: 10.1371/journal.pone.0238080 32834009 PMC7446910

[8] Jones NP. Acute Posterior Multifocal Placoid Pigment Epitheliopathy. Br J Ophthalmol (1995) 79:384–9. doi: 10.1136/bjo.79.4.384 PMC505108 7742290

[9] von Szily A. An Experimental Endogenous Transmission of Infection From Bulbus to Bulbus. Klin Monatsbl Augenheilkd (1924) 75:593–602.

[10] Krey HF Ludwig H Boschek CB. Multifocal Retinopathy in Borna Disease Virus Infected Rabbits. Am J Ophthalmol (1979) 87:157–64. doi: 10.1016/0002-9394(79)90135-1 434068

[11] Holland GN Togni BI Briones OC Dawson CR. A Microscopic Study of Herpes Simplex Virus Retinopathy in Mice. Invest Ophthalmol Vis Sci (1987) 28:1181–90. 3596994

[12] Whittum JA McCulley JP Niederkorn JY Streilein JW. Ocular Disease Induced in Mice by Anterior Chamber Inoculation of Herpes Simplex Virus. Invest Ophthalmol Vis Sci (1984) 25:1065–73. 6469490

[13] Pettit TH Kimura SJ Uchida Y Peters H. Herpes Simplex Uveitis: An Experimental Study With the Fluorescein-Labelled Antibody Technique. Invest Ophthalmol Vis Sci (1965) 4:349–57. 14326622

[14] Peiffer J RL Dekker CD Siegel FL. Ocular Lesions in Mice Following Intracerebral Injection of Herpes Simplex Virus Type I. Invest Ophthalmol Vis Sci (1983) 24:1070–8. 6307915

[15] Whittum-Hudson J Farazdaghi M Prendergast RA. A Role for T Lymphocytes in Preventing Experimental Herpes Simplex Virus Type 1-Induced Retinitis. Invest Ophthalmol Vis Sci (1985) 26:1524–32. 3877027

[16] Whittum-Hudson JA Pepose JS. Immunologic Modulation of Virus-Induced Pathology in a Murine Model of Acute Herpetic Retinal Necrosis. Invest Ophthalmol Vis Sci (1987) 28:1541–8. 3623838

[17] Atherton SS Altman NH Streilein JW. Histopathologic Study of Herpes Virus-Induced Retinitis in Athymic BALB/c Mice: Evidence for an Immunopathogenic Process. Curr Eye Res (1989) 8:1179–92. doi: 10.3109/02713688909000043 2558848

[18] Hemady R Opremcak EM Zaltas M Berger A Foster CS. Herpes Simplex Virus Type-1 Strain Influence on Chorioretinal Disease Patterns Following Intracameral Inoculation in Igh-1 Disparate Mice. Invest Ophthalmol Vis Sci (1989) 30:1750–7. 2547732

[19] Liu Y Sakai Y Minagawa H Toh Y Ishibashi T Inomata H. Induction of Bilateral Retinal Necrosis in Mice by Unilateral Intracameral Inoculation of a Glycoprotein-C Deficient Clinical Isolate of Herpes Simplex Virus Type 1. Arch Virol (1993) 129:105–18. doi: 10.1007/BF01316888 8385909

[20] Vann VR Atherton SS. Neural Spread of Herpes Simplex Virus After Anterior Chamber Inoculation. Invest Ophthalmol Vis Sci (1991) 32:2462–72. 1714427

[21] Gass JDM. Acute Posterior Multifocal Placoid Pigment Epitheliopathy. Arch Ophthalmol (1968) 80:177–85. doi: 10.1001/archopht.1968.00980050179005 5661882

[22] Floegel I Haas A El-Shabrawi Y. Acute Multifocal Placoid Pigment Epitheliopathy-Like Lesion as an Early Presentation of Subacute Sclerosing Panencephalitis. Am J Ophthalmol (2003) 135:103–5. doi: 10.1016/S0002-9394(02)01849-4 12504712

[23] Borruat FX Piguet B Herbort CP. Acute Posterior Multifocal Placoid Pigment Epitheliopathy Following Mumps. Ocul Immunol Inflammation (1998) 6:189–93. doi: 10.1076/ocii.6.3.189.4038 9785610

[24] Goldhardt R Patel H Davis JL. Acute Posterior Multifocal Placoid Pigment Epitheliopathy Following Dengue Fever: A New Association for an Old Disease. Ocul Immunol Inflammation (2016) 24:610–4. doi: 10.3109/09273948.2015.1125513 26902823

[25] Pachner AR. Neurologic Manifestations of Lyme Disease, the New “Great Imitator.” Clin Infect Dis (1989) 11:S1482–6. doi: 10.1093/clinids/11.Supplement_6.S1482 2682960

[26] Wolf M Folk J Nelson J Peeples M. Acute Posterior Multifocal Placoid Pigment Epitheliopathy and Lyme Disease. Arch Ophthalmol (1992) 110:750–0. doi: 10.1001/archopht.1992.01080180020004 1596215

[27] Anderson K Patel KR Webb L Dutton GN. Acute Posterior Multifocal Placoid Pigment Epitheliopathy Associated With Pulmonary Tuberculosis. Br J Ophthalmol (1996) 80:186–6. doi: 10.1136/bjo.80.2.186 PMC505416 8814755

[28] Randall PJ Hsu N-J Lang D Cooper S Sebesho B Allie N. Neurons Are Host Cells for Mycobacterium Tuberculosis. Infect Immun (2014) 82:1880–90. doi: 10.1128/IAI.00474-13 PMC3993430 24566619

[29] Wolf MD Folk JC Panknen CA Goeken NE. HLA-B7 and HLA-DR2 Antigens and Acute Posterior Multifocal Placoid Pigment Epitheliopathy. Arch Ophthalmol Chic Ill 1960 (1990) 108:698–700. doi: 10.1001/archopht.1990.01070070084040 2334328

[30] Kasuga I Hogg JC Paré PD Hayashi S Sedgwick EG Ruan J. Role of Genetic Susceptibility to Latent Adenoviral Infection and Decreased Lung Function. Respir Med (2009) 103:1672–80. doi: 10.1016/j.rmed.2009.05.008 PMC2757510 19502044

[31] Wolf M Folk J Goeken N. Acute Posterior Multifocal Pigment Epitheliopathy and Optic Neuritis in a Family. Am J Ophthalmol (1990) 110:89–90. doi: 10.1016/S0002-9394(14)76946-6 2368829

[32] Yeh S Lew JC Wong WT Nussenblatt RB. Relentless Placoid Chorioretinitis Associated With Central Nervous System Lesions Treated With Mycophenolate Mofetil. Arch Ophthalmol (2009) 127:341–3. doi: 10.1001/archophthalmol.2009.12 19273806

[33] Baunbæk Egelund G Ertner G Langholz Kristensen K Vestergaard Jensen A Benfield TL Brandt CT. Cerebrospinal Fluid Pleocytosis in Infectious and Noninfectious Central Nervous System Disease: A Retrospective Cohort Study. Med (Baltimore) (2017) 96:e6686. doi: 10.1097/MD.0000000000006686 PMC5419909 28471963

[34] Gonome T Suzuki Y Metoki T Takahashi S Nakazawa M. Acute Posterior Multifocal Placoid Pigment Epitheliopathy and Granulomatous Uveitis Following Influenza Vaccination. Am J Ophthalmol Case Rep (2016) 4:60–3. doi: 10.1016/j.ajoc.2016.08.008 PMC5757482 29503928

[35] Mendrinos E Baglivo E. Acute Posterior Multifocal Placoid Pigment Epitheliopathy Following Influenza Vaccination. Eye (2010) 24:180–1. doi: 10.1038/eye.2009.68 19343055

[36] Tanaka H Park C-H Ninomiya A Ozaki H Takada A Umemura T. Neurotropism of the 1997 Hong Kong H5N1 Influenza Virus in Mice. Vet Microbiol (2003) 95:1–13. doi: 10.1016/S0378-1135(03)00132-9 12860072

[37] van Riel D Leijten LM Verdijk RM GeurtsvanKessel C van der Vries E van Rossum AMC. Evidence for Influenza Virus CNS Invasion Along the Olfactory Route in an Immunocompromised Infant. J Infect Dis (2014) 210:419–23. doi: 10.1093/infdis/jiu097 24550441

[38] Ashfaq I Vrahimi M Waugh S Soomro T Grinton ME Browning AC. Acute Macular Neuroretinopathy Associated With Acute Influenza Virus Infection. Ocul Immunol Inflammation (2021) 29:333–9. doi: 10.1080/09273948.2019.1681470 31697568

[39] McWilliams IL Kielczewski JL Ireland DDC Sykes JS Lewkowicz AP Konduru K. Pseudovirus RvsvΔg-ZEBOV-GP Infects Neurons in Retina and CNS, Causing Apoptosis and Neurodegeneration in Neonatal Mice. Cell Rep (2019) 26:1718–1726.e4. doi: 10.1016/j.celrep.2019.01.069 30759384 PMC6748882

[40] Jones BE Jampol LM Yannuzzi LA Tittl M Johnson MW Han DP. Relentless Placoid Chorioretinitis - A New Entity or an Unusual Variant of Serpiginous Chorioretinitis? Arch Ophthalmol (2000) 118:931–8. doi: 10-1001/pubs.Ophthalmol.-ISSN-0003-9950-118-7-ecs80142 10900106

[41] Taylor MP Enquist LW. Axonal Spread of Neuroinvasive Viral Infections. Trends Microbiol (2015) 23:283–8. doi: 10.1016/j.tim.2015.01.002 PMC4417403 25639651

[42] Kramer T Enquist LW. Alphaherpesvirus Infection Disrupts Mitochondrial Transport in Neurons. Cell Host Microbe (2012) 11:504–14. doi: 10.1016/j.chom.2012.03.005 PMC3358700 22607803

[43] To Spread, Nervous System Viruses Sabotage Cell, Hijack Transportation. Available at: https://www.princeton.edu/news/2012/05/30/spread-nervous-system-viruses-sabotage-cell-hijack-transportation (Accessed October 26, 2021).

[44] Beirowski B Nógrádi A Babetto E Garcia-Alias G Coleman MP. Mechanisms of Axonal Spheroid Formation in Central Nervous System Wallerian Degeneration. J Neuropathol Exp Neurol (2010) 69:455–72. doi: 10.1097/NEN.0b013e3181da84db 20418780

[45] Rajaee A Geisen ME Sellers AK Stirling DP. Repeat Intravital Imaging of the Murine Spinal Cord Reveals Degenerative and Reparative Responses of Spinal Axons in Real-Time Following a Contusive SCI. Exp Neurol (2020) 327:113258. doi: 10.1016/j.expneurol.2020.113258 32105708 PMC7549695

[46] Klufas MA Phasukkijwatana N Iafe NA Prasad PS Agarwal A Gupta V. Optical Coherence Tomography Angiography Reveals Choriocapillaris Flow Reduction in Placoid Chorioretinitis. Ophthalmol Retina (2017) 1:77–91. doi: 10.1016/j.oret.2016.08.008 31047399

[47] Jampol LM. Multiple Evanescent White Dot Syndrome: I. Clinical Findings. Arch Ophthalmol (1984) 102:671. doi: 10.1001/archopht.1984.01040030527008 6721749

[48] Moorthy RS Inomata H Rao NA. Vogt-Koyanagi-Harada Syndrome. Surv Ophthalmol (1995) 39:265–92. doi: 10.1016/s0039-6257(05)80105-5 7725227

[49] Bhavsar KV Lin S Rahimy E Joseph A Freund KB Sarraf D. Acute Macular Neuroretinopathy: A Comprehensive Review of the Literature. Surv Ophthalmol (2016) 61:538–65. doi: 10.1016/j.survophthal.2016.03.003 26973287

[50] Yang C-S Hsieh M-H Su H-I Kuo Y-S. Multiple Evanescent White Dot Syndrome Following Acute Epstein-Barr Virus Infection. Ocul Immunol Inflammation (2019) 27:244–50. doi: 10.1080/09273948.2017.1371763 29020489

[51] Bassili SS Peyman GA Gebhardt BM Daun M Ganiban GJ Rifai A. Detection of Epstein-Barr Virus DNA by Polymerase Chain Reaction in the Vitreous From a Patient With Vogt-Koyanagi-Harada Syndrome. Retina Phila Pa (1996) 16:160–1. doi: 10.1097/00006982-199616020-00013 8724962

[52] Usui M Usui N Goto H Sakai J Osato T. Detection of Epstein-Barr-Virus DNA by Polymerase Chain-Reaction in Cerebrospinal Fluid From Patients With Vogt-Koyanagi-Harada Disease. Invest Ophthalmol Vis Sci (1991) 32:807–7.

[53] Aoyagi R Hayashi T Masai A Mitooka K Gekka T Kozaki K. Subfoveal Choroidal Thickness in Multiple Evanescent White Dot Syndrome. Clin Exp Optom (2012) 95:212–7. doi: 10.1111/j.1444-0938.2011.00668.x 22023216

[54] Dodwell DG Jampol LM Rosenberg M Berman A Zaret CR. Optic Nerve Involvement Associated With the Multiple Evanescent White-Dot Syndrome. Ophthalmology (1990) 97:862–8. doi: 10.1016/S0161-6420(90)32489-2 2381699

[55] Maruko I Iida T Sugano Y Oyamada H Sekiryu T Fujiwara T. Subfoveal Choroidal Thickness After Treatment Of Vogt-Koyanagi-Harada Disease. Retina- J Retin Vitr Dis (2011) 31:510–7. doi: 10.1097/IAE.0b013e3181eef053 20948460

[56] Nakao K Abematsu N Mizushima Y Sakamoto T. Optic Disc Swelling in Vogt-Koyanagi-Harada Disease. Invest Ophthalmol Vis Sci (2012) 53:1917–22. doi: 10.1167/iovs.11-8984 22408010

[57] Hashimoto Y Saito W Saito M Hasegawa Y Ishida S. Increased Thickness and Decreased Blood Flow Velocity of the Choroid in a Patient With Acute Macular Neuroretinopathy. BMC Ophthalmol (2019) 19:109. doi: 10.1186/s12886-019-1123-0 31088423 PMC6518730

[58] Shah GK Cooper BA Grand MG Hart WM. Acute Macular Neuroretinopathy and Associated Disc Swelling and Blind Spot Enlargement. Can J Ophthalmol (2003) 38:602–4. doi: 10.1016/S0008-4182(03)80116-3 14740804

[59] Hirooka K Saito W Hashimoto Y Saito M Ishida S. Increased Macular Choroidal Blood Flow Velocity and Decreased Choroidal Thickness With Regression of Punctate Inner Choroidopathy. BMC Ophthalmol (2014) 14:73. doi: 10.1186/1471-2415-14-73 24885365 PMC4041897

[60] Zhang X Zuo C Li M Chen H Huang S Wen F. Spectral-Domain Optical Coherence Tomographic Findings at Each Stage of Punctate Inner Choroidopathy. Ophthalmology (2013) 120:2678–83. doi: 10.1016/j.ophtha.2013.05.012 23769333

[61] Ramtohul P Gascon P Denis D. Outer Retinal Plume Signature in Multiple Evanescent White Dot Syndrome. Ophthalmol Retina (2020) 4:766. doi: 10.1016/j.oret.2020.03.023 32768030

[62] Tanigawa M Tsukahara Y Yamanaka H. A Case of Acute Posterior Multifocal Placoid Pigment Epitheliopathy Demonstrating Vogt-Koyanagi-Harada Disease-Like Optical Coherence Tomography Findings in the Acute Stage. Case Rep Ophthalmol (2013) 4:172–9. doi: 10.1159/000356051 PMC3884189 24403900

[63] Lee GE Lee BW Rao NA Fawzi AA. Spectral Domain Optical Coherence Tomography and Autofluorescence in a Case of Acute Posterior Multifocal Placoid Pigment Epitheliopathy Mimicking Vogt-Koyanagi-Harada Disease: Case Report and Review of Literature. Ocul Immunol Inflammation (2011) 19:42–7. doi: 10.3109/09273948.2010.521610 21034311

[64] Kitamura Y Oshitari T Kitahashi M Baba T Yamamoto S. Acute Posterior Multifocal Placoid Pigment Epitheliopathy Sharing Characteristic OCT Findings of Vogt-Koyanagi-Harada Disease. Case Rep Ophthalmol Med (2019) 2019:9217656. doi: 10.1155/2019/9217656 31380133 PMC6652076

[65] Almalki K Alsulaiman SM Abouammoh MA. Internal Limiting Membrane Folds as a Presenting Sign in Acute Initial-Onset Vogt-Koyanagi-Harada Disease: A Case Report. Ocul Immunol Inflammation (2020), 1–5. doi: 10.1080/09273948.2020.1828489 33054464

[66] Long CP Bakhoum MF Freeman WR. Depigmented Chorioretinal Lesions Following Varicella-Zoster Virus Infection. JAMA Ophthalmol (2020) 138:e201652. doi: 10.1001/jamaophthalmol.2020.1652 33180134

[67] Hareesh Reddy LS Bhandary VS Rao AK Rao GL. Unilateral Multifocal Chorioretinopathy a Clue to Herpes Zoster Ophthalmicus. J Evol Med Dent Sci (2016) 5:4596–7. doi: 10.14260/jemds/2016/1047

[68] Steptoe PJ Scott JT Baxter JM Parkes CK Dwivedi R Czanner G. Novel Retinal Lesion in Ebola Survivors, Sierra Leone, 2016. Emerg Infect Dis (2017) 23:1102–9. doi: 10.3201/eid2307.161608 PMC5512503 28628441

[69] Steptoe PJ Momorie F Fornah AD Komba SP Emsley E Scott JT. Multimodal Imaging and Spatial Analysis of Ebola Retinal Lesions in 14 Survivors of Ebola Virus Disease. JAMA Ophthalmol (2018) 136:689. doi: 10.1001/jamaophthalmol.2018.1248 29800941 PMC6542654

[70] Khairallah M Ben Yahia S Attia S Zaouali S Ladjimi A Messaoud R. Linear Pattern of West Nile Virus-Associated Chorioretinitis is Related to Retinal Nerve Fibres Organization. Eye (2007) 21:952–5. doi: 10.1038/sj.eye.6702355 16628235

[71] Khairallah M Benyahia S Ladjimi A Zeghidi H Benromdhane F Besbes L. Chorioretinal Involvement in Patients With West Nile Virus Infection☆. Ophthalmology (2004) 111:2065–70. doi: 10.1016/j.ophtha.2004.03.032 15522373

[72] Doan T Acharya NR Pinsky BA Sahoo MK Chow ED Banaei N. Metagenomic DNA Sequencing for the Diagnosis of Intraocular Infections. Ophthalmology (2017) 124:1247–8. doi: 10.1016/j.ophtha.2017.03.045 PMC5533302 28526549

