# Supplementary figures and images for: Proposing a Neurotropic Etiology for Acute Posterior Multifocal Placoid Pigment Epitheliopathy and Relentless Placoid Chorioretinitis
Source: Front Ophthalmol (Lausanne). 2022 Jan 10;1:802962. doi: 10.3389/fopht.2021.802962 (PMC11182168; doi:10.3389/fopht.2021.802962)

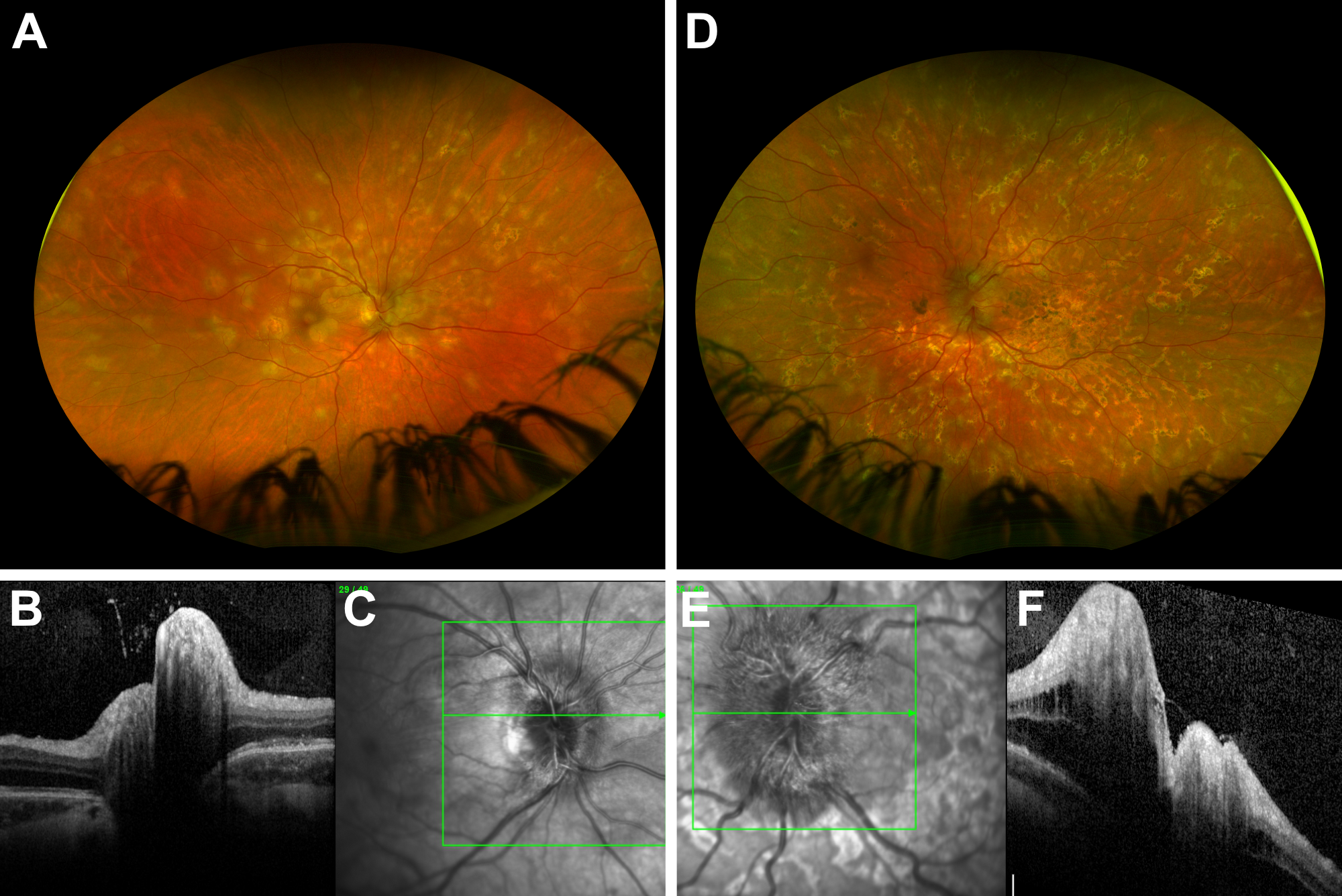

Supplement: Supplementary Figure 1 — Disc edema proportional to lesion quantity. (A). Right eye ultra-widefield fundus imaging, 74 days post symptom onset. Comparatively fewer lesions than the left eye seen in ultra-wide field fundus image D. (B). Right eye optic disc spectral-domain optical coherence tomography (SD-OCT) demonstrating optic disc edema but significantly less edema in comparison to the left eye (E, F). (C). Right eye optic disc infrared (IR) image. (D). Left eye ultra-widefield fundus image. (E). Left eye optic disc IR image. (F). Left eye optic disc SD-OCT. [file Image_1.tiff]

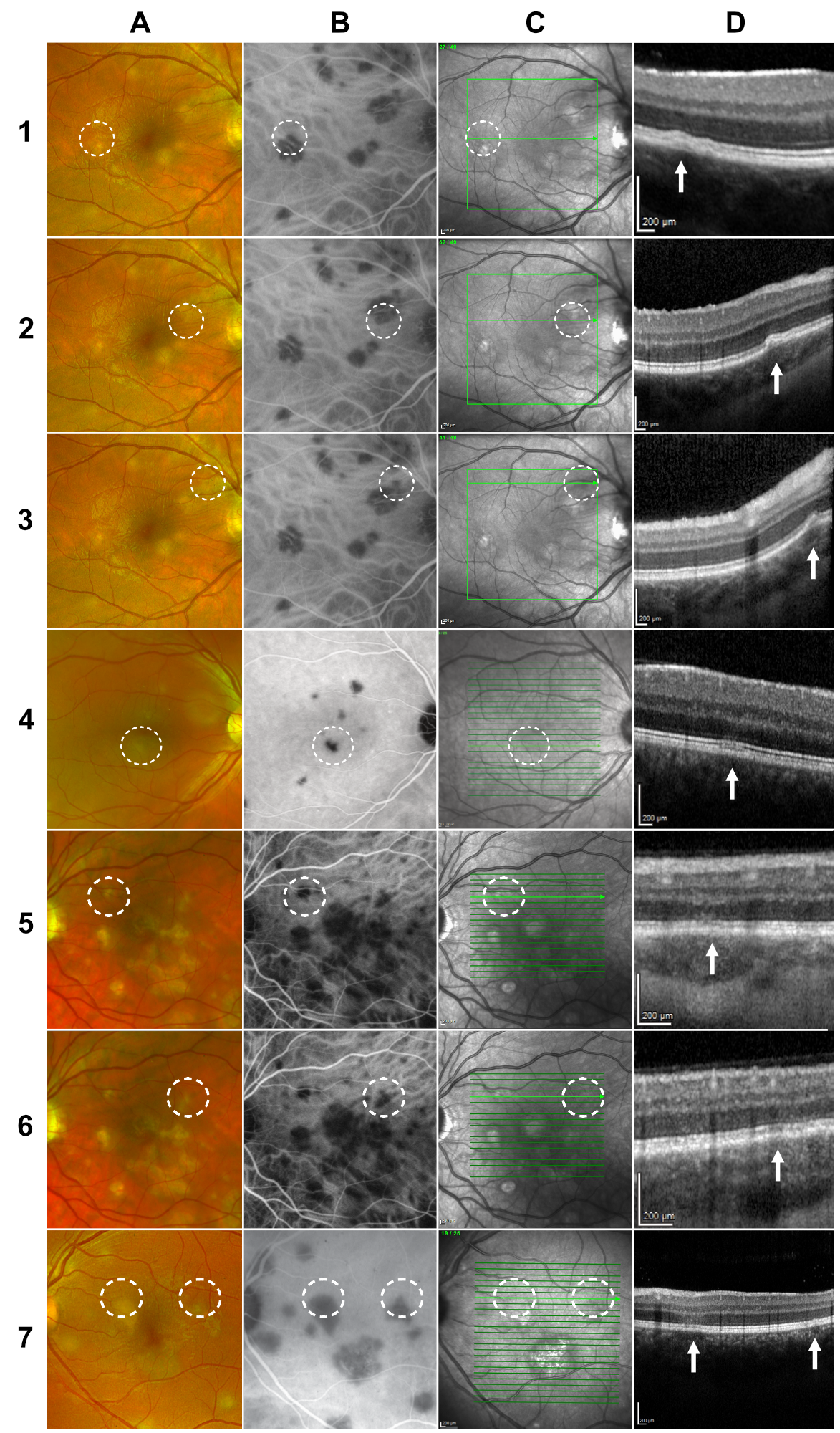

Supplement: Supplementary Figure 2 — Multimodal imaging comparison of early lesions. Column A, Fundus scanning laser ophthalmoscopy appearance. Column B, Corresponding hypofluorescence seen on indocyanine green angiography. Column C, Infrared fundus imaging. Column D, Spectral-domain optical coherence tomography (SD-OCT) demonstrating acute lesions with retinal pigment epithelium and Bruch’s membrane separation. White circles indicate the lesion location on comparative modalities. White arrows indicate location of lesions on SD-OCT. [file Image_2.tiff]

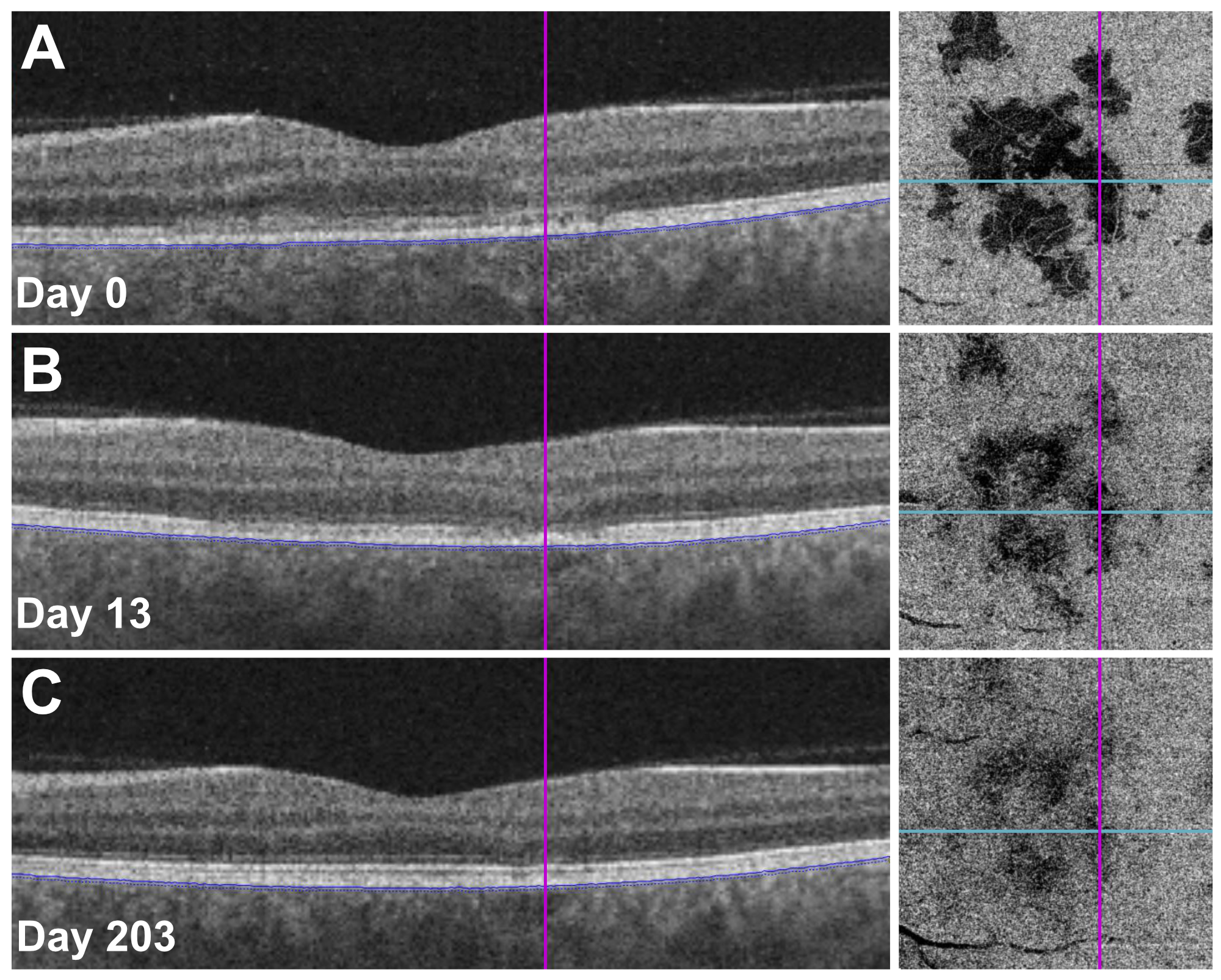

Supplement: Supplementary Figure 3 — APMPPE lesion recovery. (A). Swept-source optical coherence tomography (SS-OCT) of acute lesion with outer nuclear layer (ONL) hyperreflectivity. (B). Corresponding en face OCT angiography (OCTA) demonstrating associated choriocapillaris hypoperfusion. (C). Appearance at day 13 demonstrating resolution of the ONL hyperreflectivity with loss of the ellipsoid zone (EZ) and interdigitation zone (IDZ) with a dipping of overlying structures. (D). Partial resolution of choriocapillaris perfusion on OCTA. (E). Appearance at day 203, demonstrating outer retinal structure restoration. (F). Corresponding improvement of choriocapillaris perfusion on OCTA. Purple line denotes lesion location on SS-OCT. Purple and turquoise intersection denote corresponding APMPPE lesion location on en face OCTA imaging. [file Image_3.tiff]

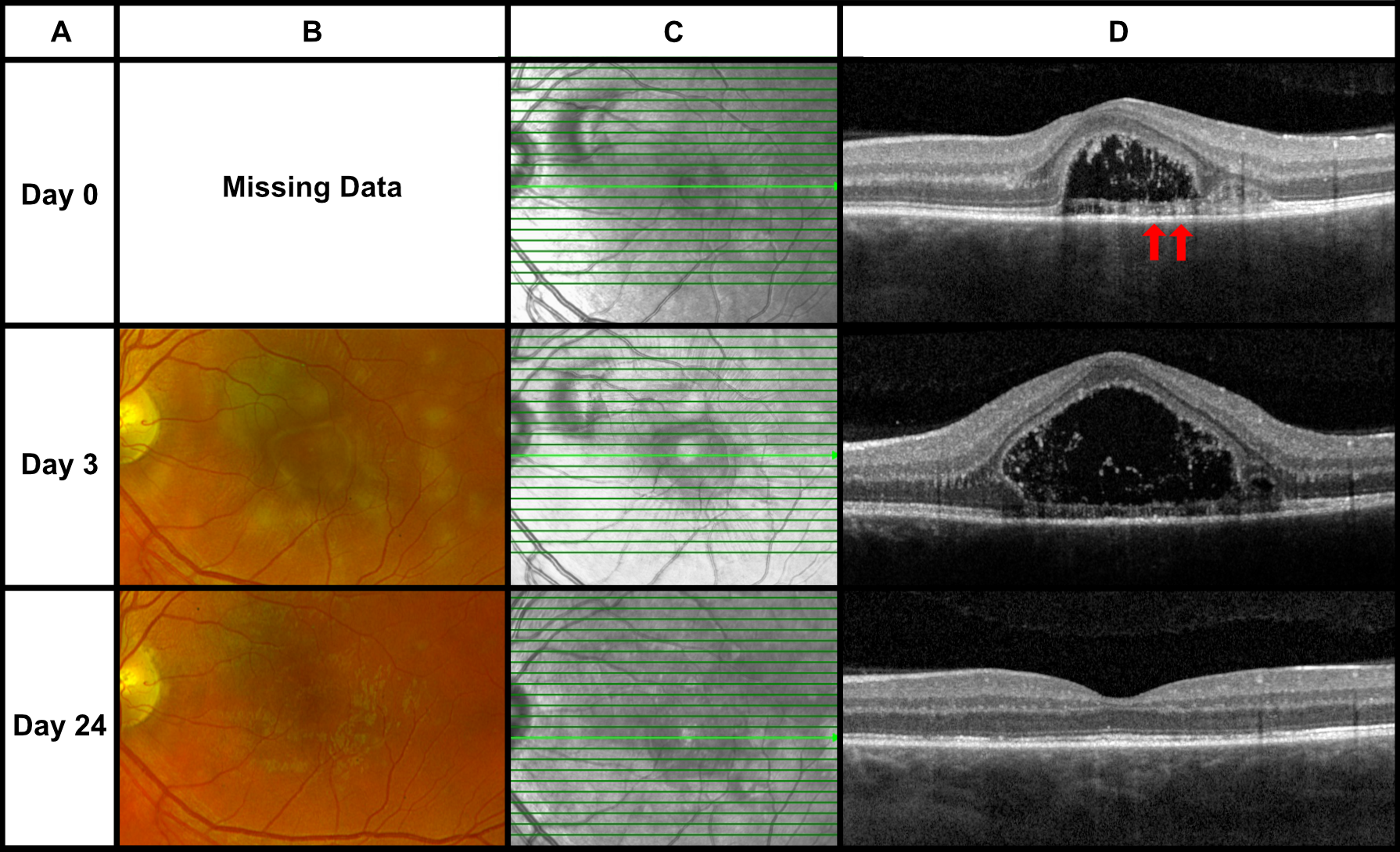

Supplement: Supplementary Figure 4 — Case 4, Left eye. (A), Days since presentation. (B), Scanning laser ophthalmoscopy (C), Infrared fundus image. Bold green line denotes spectral-domain optical coherence tomography (SD-OCT) scan position. (D), SD-OCT. Creamy colored acute lesions are seen on color fundus imaging at day 3 and fade by day 24. SD-OCT demonstrates a bacillary layer detachment of two adjacent lesions which coalesce by day 3 with underlying separation of the retinal pigment epithelium (RPE) from Bruch’s membrane (BM) observed at presentation (Red arrows). Bacillary layer detachment worsens acutely following presentation before resolving without treatment by day 24. Presenting Snellen visual acuity was 6/18 which decreased to 6/24 by day 3 but improved to 6/9 by day 24 and 6/6 by the following review at day 85. [file Image_4.tiff]

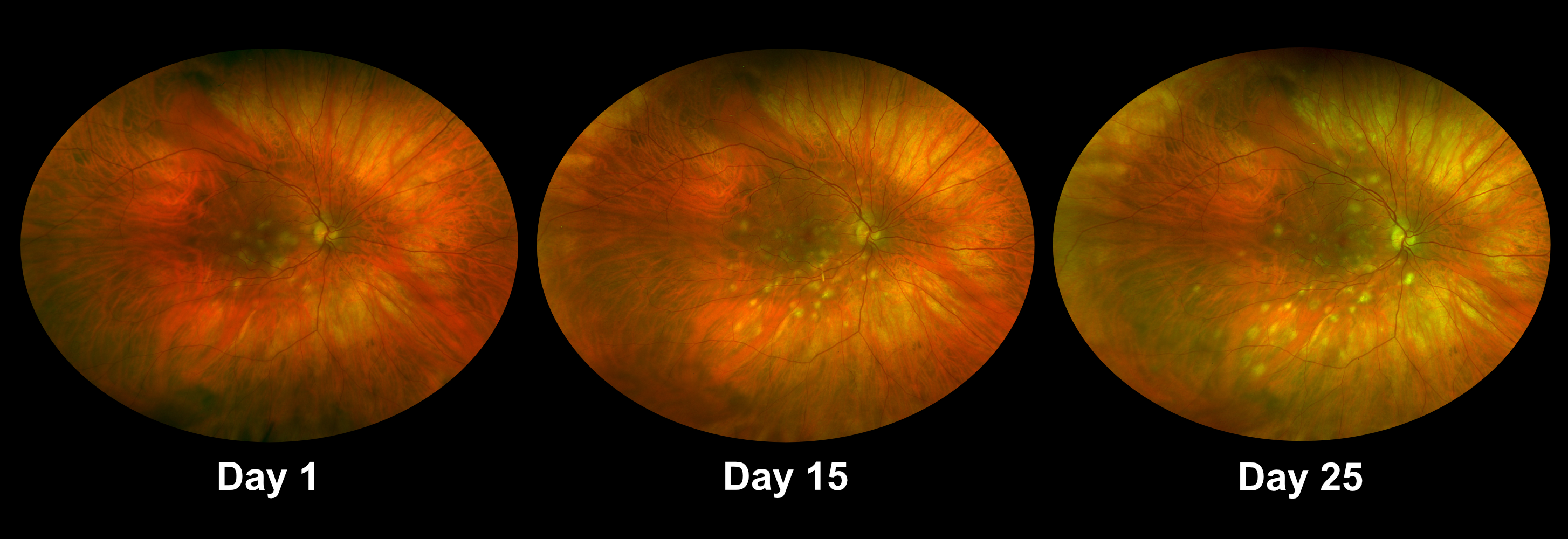

Supplement: Supplementary Figure 5 — Acute posterior multifocal placoid pigment epitheliopathy (APMPPE) resembling Birdshot Chorioretinopathy, Right eye. Initial presentation demonstrated multifocal APMPPE lesions within the macula. By days 15 and 25, central lesions appear to fade as new lesions radiate towards the peripheral retina. The patient was HLA A29 negative. [file Image_5.tiff]
